# A 3D Printed Vitrification Device for Storage in Cryopreservation Vials

**DOI:** 10.3390/app11177977

**Published:** 2021-08-28

**Authors:** Yue Liu, Andy Lin, Terrence R. Tiersch, William Todd Monroe

**Affiliations:** 1 Department of Biological and Agricultural Engineering, Louisiana State University Agricultural Center, Louisiana State University, Baton Rouge, LA 70803, USA; 2 Aquatic Germplasm and Genetic Resources Center, School of Renewable Natural Resources, Louisiana State University Agricultural Center, Baton Rouge, LA 70820, USA

**Keywords:** vitrification, device, 3D printing, sperm cryopreservation, open hardware, standardization

## Abstract

Sperm cryopreservation by vitrification is a promising approach for small-bodied animals such as zebrafish (*Danio rerio*). However, most vitrification tools adopted in aquatic research were initially designed for applications other than sperm (such as human embryo freezing) and, thus, pose challenges for adoption to sperm vitrification. Three-dimensional (3D) printing combined with open hardware sharing is an emerging strategy to address challenges in the development of cryopreservation tools. The goal of this study was to develop a 3D printed Vitrification Device for Cryo-Vials (VDCV) that can be integrated with the existing vial storage systems. The VDCV combined the vitrification and handling components to achieve functions of sample handling, vitrification, storage, and identification. The vitrification component featured a base, a stem, and a loop. A total of 36 configurations with various loop lengths (8, 10, and 12 mm); loop widths (2.0, 2.5, 3.0, and 3.5 mm); and support structures (open, transverse, and axial) of the VDCD prototypes were designed, fabricated, and tested. Device handling orientations (horizontal and vertical holding angles prior to and during freezing) were also investigated. Computer simulations estimated that the cooling rate of the samples ranged from 0.6–1.5 × 10^5^ °C/min in all the configurations. Prior to freezing, loops with axial supports produced a minimum of 92% film retention. The overall trends of full vitrification occurrence were observed: horizontal plunging > vertical plunging, and axial support > transverse support and open loop. A loop length of 8 mm had the highest overall vitrification occurrence (86–100%). No significant differences (*p* = 0.6584) were shown in a volume capacity (5.7–6.0 μL) among the three supporting configurations. A single unit of VDCV can provide loading efficiencies of about 6 × 10^7^ sperm/vial, pooling of samples from 3–6 males/vial, and fertilization for 1800 eggs/vial. The VDCV are low-cost (<$0.5 material cost per unit) and can be customized, standardized, securely labeled, and efficiently stored. The prototypes can be accessed by user communities through open-fabrication file sharing and fabricated with consumer-level 3D printers, thus facilitating community-level standardization.

## 1. Introduction

Sperm cryopreservation is the core process in the development of germplasm repositories to assist in the genetic improvement of domestic animals, conservation of endangered animals, and biomedical research of model species. There are two major sperm cryopreservation approaches. Conventional cryopreservation (‘equilibrium freezing’ or ‘slow cooling’) requires identifying and achieving the ideal cooling rates (e.g., 5–40 °C/min from 4 to −80 °C) that can be fast enough to minimize the toxic solution effects and slow enough to minimize intracellular ice formation [1]. This approach is ideal for processing large numbers of samples in batches, but specialized equipment can cost tens of thousands of dollars (e.g., >US$20,000 for a computer-programmed freezer). An alternative and relatively new method for sperm cryopreservation is vitrification, by which the liquid is cooled at >1000 °C/min (‘rapid cooling’) to transform into an amorphous solid (glass) phase without the formation of crystalline ice [2,3]. The rapid cooling can be obtained simply by plunging a thin film (e.g., several μL loaded on loops) or droplets (e.g., on plates or strips) of the sample into liquid nitrogen. Vitrification has several benefits over equilibrium freezing, including low cost, portability, and superior post-thaw sample quality [4,5]. It is suitable for small sample volumes (e.g., <100 μL) or small-scale freezing for research purposes.

The development of inexpensive and varied sperm vitrification tools has been slow and hampers the wide application and research of this technology. Commercial sperm vitrification products are scarce. The Sperm VD device [6] designed for sperm vitrification with storing and labeling mechanisms costs $74 each (https://us.ivfstore.com/products/sperm-vd?variant=12851114180685, accessed on 27 August 2021) and can only accommodate about 1 μL of sperm sample per device. Most sperm vitrification studies have been constrained to the adoption of tools that were initially designed for applications other than sperm, such as the Cryoloop (Hampton Research, Aliso Viejo, CA, USA) originally designed for crystallography [7,8], the Cryotop^®^ (KITAZATO, Valencia, Spain) designed for the vitrification of human oocytes and embryos [9,10], pipette tips for liquid transfer [11], cryopreservation straws (IMV Technologies, L’Aigle, France) for the equilibrium freezing of sperm [12], and inoculation loops for microbiology [11]. Although these tools can be used for vitrification purposes, they can be expensive (e.g., the Cryotop^®^ costs more than $20/device for 2-μL samples) and are difficult to redesign, customize, securely label, and efficiently store.

Three-dimensional (3D) printing combined with open-source sharing mechanisms is a promising strategy to address challenges in the development of sperm vitrification hardware. A version of this strategy has been successfully applied in software development and now is fueling a new movement in scientific hardware development [13]. Open hardware sharing allows maker community members to gain access to hardware designs through online platforms for free. Thanks to the increasing accessibility of 3D printers, user community members can download, fabricate, and assemble designs and devices for a low cost instead of purchasing expensive proprietary equipment or repurposing existing tools. In addition, members of user communities and maker communities can contribute to design changes for modifications and improvements, facilitating community-level standardization.

In recent years, this potential has been explored for the application of 3D printing in cryobiology applications [14]. A 3D printed cryopreservation device was developed to freeze small batches (several dozens) of samples in French straws (0.25 mL and 0.5 mL) or cryovials (50–100 μL) with standardizable cooling rates controlled by a modular assembly with liquid nitrogen vapor in Styrofoam boxes [15] and shipping dewars [16]. A 3D printed motorized controlled cooling conveyor device (CCCD) was developed for the continuous cryopreservation of non-batched sperm samples [17]. The feasibility of 3D printing for sperm vitrification application was demonstrated with simple 3D printed vitrification configurations [18,19] featuring a single-piece loop that could load the sperm samples in the form of thin films. Based on this prototyping, an upgraded 3D printed vitrification device was developed with practical functions, including vitrification, volume control, labeling, protection, and storage within the existing straw storage systems [20]. All of these designs addressed the main problems of film formation and stability, rapid heat transfer to accommodate vitrification, permanent labeling, and protection and storage of the delicate devices and frozen films.

In addition to French straws, another widely applied type of cryopreservation containers are vials with liquid-tight lids [21,22]. A variety of commercially available vials have been developed to enable the high-throughput and efficient management of cryopreserved samples. To accommodate storage in vials, wider loops can be designed beyond the dimensions of the aforementioned studies (that were constrained by the width of 0.5-mL straws). The goal of this study was to develop a 3D printed Vitrification Device for Cryo-Vials (VDCV) that can be integrated with existing cryo-vial storage systems. The specific objectives were to: (1) design and fabricate the VDCV, (2) evaluate the cooling rates by computer simulation, (3) evaluate the sample loading methods prior to freezing, (4) evaluate the vitrification occurrence, and (5) determine the volume capacity. The initial progress in the present study provides a foundation and guidance for further community-driven efforts in the development of vitrification devices with efficient storage systems.

## 2. Materials and Methods

### 2.1. Design and Fabrication of Prototypes with Various Dimensions

**Figure 1.**
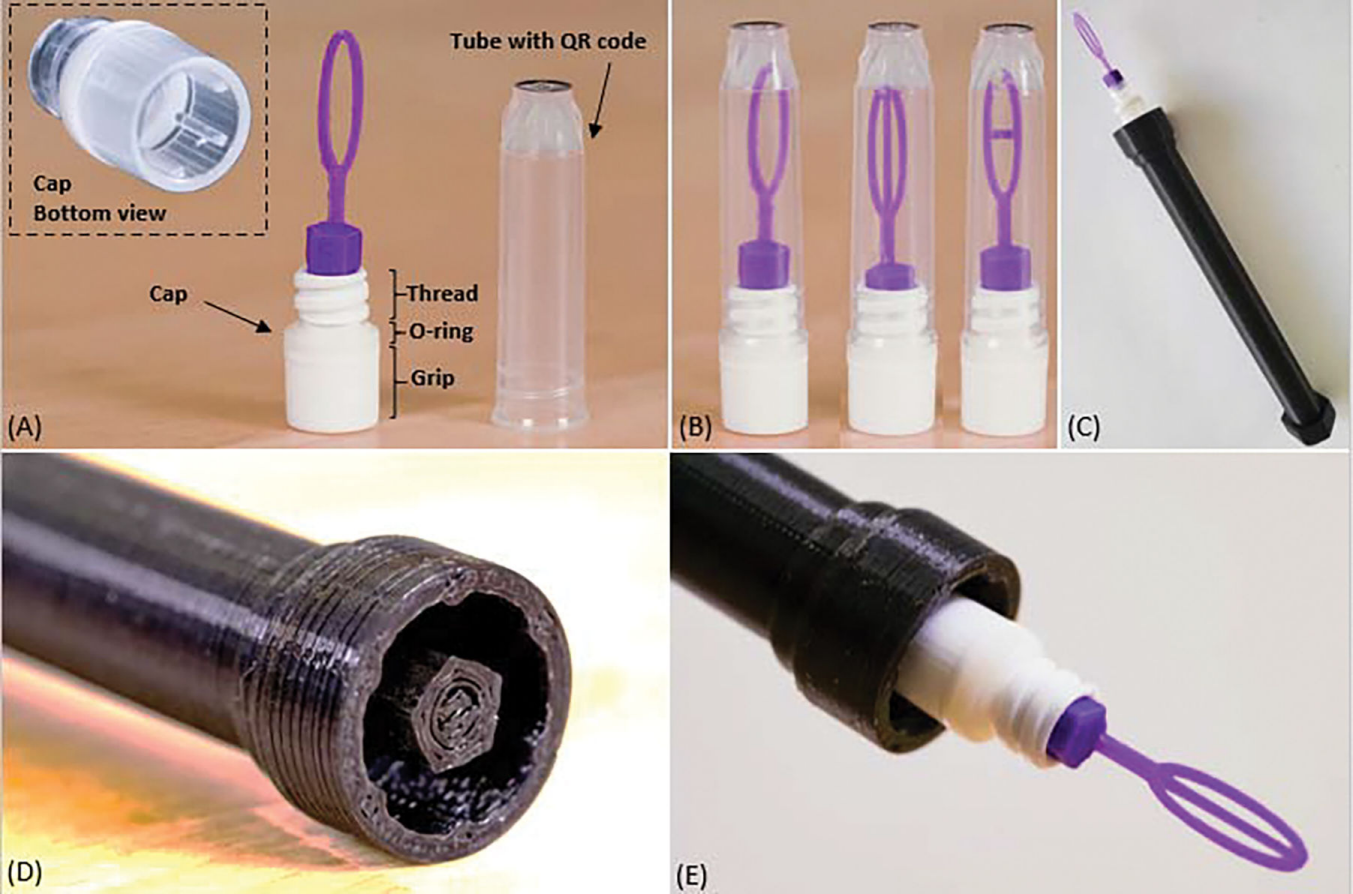
Vitrification Device for Cryo-Vials (VDCV) fabricated by 3D printing. (**A**) A 0.5-mL Nunc^™^ Bank-It^™^ cryogenic storage vial comprises a cap and a tube. (**B**) Vitrification components with three different configurations (from left to right): open loop, axial support, and transverse support. A handling component (**C**) with structures (**D**) to fit 0.5-mL Bank-It^™^ cryogenic storage vials (**E**).

The overall design goal was to create a device that could suspend a thin film of sperm sample within a loop that could fit into Nunc^™^ Bank-It^™^ cryogenic storage vials (Thermo Fisher Scientific, Waltham, MA, USA) for sample storage and management. These vials are routinely used in cooling and storage of sperm at the Zebrafish International Resource Center (ZIRC) and other research laboratories (personal communication with ZIRC). Each unit of vial (Figure 1A) featured a tube with QR codes at the bottom and a cap with an inner hollow thread for fastening inside the tube, an O-ring for sealing, and an outer hollow grip at the bottom for handling and mounting of the decapping tools. Two components were designed for the VDCV: a vitrification component (Figure 1B) with a loop to form thin films and a handling component (Figure 1C) with a cylindrical shaft to facilitate sample processing (Figure 1D,E). The design considerations are detailed below. The vitrification component should fit in 0.5-mL Bank-It^™^ vial tubes with an internal diameter of 6.8 mm and length of 20 mm and could be fastened inside the hollow thread (with an inner diameter of 4.8 mm). The handle should be fastened to the hollow grip (with an inner diameter of 6.7 mm). Based on the preliminary trials of sample loading and vitrification feasibility, the loops of the vitrification component were designed as three lengths (8, 10, and 12 mm) of the major axis (referred to as ‘loop lengths’) and four lengths (2, 2.5, 3, and 3.5 mm) of the minor axis (referred to as ‘loop widths’). Previously reported vitrification devices had a loop width of 2.1 mm to accommodate storage within straw sleeves [20]. Because the higher loop widths were designed in the present study to take advantage of a larger cross-sectional area of vials, support mechanisms were evaluated to stabilize films prior to freezing. Within the loop, three variations of film support configurations were designed: open loop, loop with axial support, and loop with transverse support. A total of 36 configurations (3 loop lengths × 4 loop widths × 3 supports) were developed for operational testing.

To support the open-fabrication availability of designs, consumer-level software and 3D printers were used. Designs of 3D modeling were created using a free online platform for Computer-Assisted Design (CAD) (Onshape, PTC Inc., Boston, MA, USA). The 3D renderings of the prototypes were converted to stereolithography (STL) files and imported into free slicing software (Ultimaker Cura V4.6, Ultimaker, Utrecht, The Netherlands) to define the printer settings (Supplemental Table S1) in G-Code format. The settings were loaded onto a fused deposition modeling (FDM) 3D printer (SOVOL, Sv01, SOVOL 3D^®^, NOVA SILK ROAD SARL, Paris, France) printed with a PLA filament (ZYLtech Engineering, Houston, TX, USA). For a batch of 5 duplicates (Figure S1) of each vitrification component, printing took 8–15 min and <US$0.01 (~0.4 g of PLA filament). The handling component took 1.5–2 h and <US$0.5 (~10 g) to print. As such, about US$0.5 material cost was required to print a the handling component of the VDCV and an additional US$0.5 to print 250 pieces of the vitrification components (loops).

### 2.2. Comparison of Designs with Computer Simulation

To assist in CVCD support configuration design and facilitate prototyping, computational fluid dynamics (CFD) software (Autodesk CFD 2019, Autodesk, San Rafael, CA, USA) was used to compare the cooling rates and temperature distributions of designs with different loop support configurations. The 3D models of the vitrification loops were created in Autodesk Inventor (Autodesk), converted to STL files, and imported to Autodesk CFD. The following material properties were assumed to not vary with the temperature and were assigned for polylactic acid (PLA) based on the manufacturer’s specifications, including 0.13-W/m thermal conductivity, 1.29-g/cm^3^ density, 1800-J/kg^−k^ specific heat, and 0.92 emissivity. The initial temperature of the loops was set at 20 °C to simulate room temperature prior to plunging into liquid nitrogen. A boundary condition of 200 °C (liquid nitrogen temperature) steady-state temperature was assigned to all surfaces to simulate the temperature during exposure (e.g., rapid plunging) to liquid nitrogen. Automatic mesh sizing was initially uniformly applied and refined in the size adjustment from the default 1.0 to 0.2. The meshing element count for each design was above 1 million elements.

To facilitate the computations, more complicated physical phenomena, such as phase transition, surface tension, and convection, were not considered in this study. Heat transfer models were applied, and the flow dynamics were disabled. The simulation process was run in a transient mode, with time steps of 0.001 s and an inner interval of ‘1’. The cooling rate was calculated as the time (min) to cool water to from 20 °C to −137 °C (136 K, the commonly acknowledged glass transition temperature of water) [23]. The physical properties of water were assigned to the films within loop structures. To simplify the simulation, the thickness of the films was set at 0.4 mm (flush with the upper and bottom surfaces of the loops) without taking into consideration the surface curvature caused by meniscus effects, liquid surface tension, and gravity. The cooling rates at 5 positions in the open loop design were sampled in the simulations, including the center position for open loops (PO), centers of the two compartments divided by axial supports (PA1 and PA2), and centers of the two compartments divided by transverse supports (PT1 and PT2). Cooling rates were averaged for PA1 and PA2 as PA and PT1 and PT2 as PT.

### 2.3. Evaluation of the Effects of Sample Handling Methods and Device Configurations on Film Retention Rates

Different sample loading methods and loop configurations can affect the retention of sample films on the device prototypes prior to freezing. Deionized (DI) water (without cryoprotectant) was used as a model fluid in this experiment. Loops of the VDCV were submerged vertically into DI water for >2 s. Upon removal from the liquid, the loops were held horizontally or vertically (as two different handling methods) for 5 s to observe whether the water films were retained or not at the 5th second. Film loading and observation were repeated 10 times, and the film retention rate was calculated as: (times of retention/10) × 100%. Three loop lengths and four loop widths were tested with 5 replicates (printed in a single batch) for each configuration.

### 2.4. Evaluation of the Effects of Plunging Orientation and Device Configurations on Vitrification Occurrence

Based on the film retention experiment, different orientations during the sample plunging into liquid nitrogen were evaluated for their effect on the vitrification. A 3D printed sample plunging device was developed (Figure S2) that could hold loops at horizontal or vertical orientations to the liquid nitrogen surface and standardize the distance of descent and ascent with a sliding track. To evaluate the quality of vitrification formation, a standardized visual classification system was adapted as described below [18,20]. After submerging in liquid nitrogen for about 2 s, the samples were removed and transferred to an observation station (Figure S2). The observation station was positioned in a liquid nitrogen vapor environment to extend the observation time prior to sample thawing. Frozen films in loops were precisely positioned in front of 2D barcodes and visually examined. A previous standard approach [18,19] classified the vitrification outcomes into six categories by the visibility of the barcodes: ‘No sample’, ‘Film failure’, ‘Fractured’, Zero (opaque and crystalline)’, ‘One (translucent and partially vitrified)’, and ‘Two (transparent and vitrified)’ (only ‘Zero’, ‘One’, and ‘Two’ were included in the statistical analyses). To facilitate the statistical analysis and training process, these six categories were grouped as: (1) “No vitrification’ (including ‘No sample’ and ‘Film failure’ samples); (2) ‘possible vitrification’ (including ‘Fractured’, ‘Opaque’, and ‘Translucent’ films), or (3) ‘Full vitrification’ (including transparent films). A maximum time for assessment was set at ≤3 s to ensure that the classifications were assigned before the films began to thaw. Vitrification was also evaluated with an existing vitrification solution used for aquatic species (20% Hanks’ balanced salt solution, 40% methanol, 20% methyl glycol, and 20% 1,2 propanediol) [20]. All of the loops filled with this viscous solution retained their films prior to plunging into liquid nitrogen.

To evaluate the effects of device configurations on vitrification, the three film support designs (open loop, axial support, and transverse support) were tested with loops with 10-mm lengths, and the support design with the best performance was selected to compare the effects of the three different loop lengths (8, 10, and 12 mm). The two plunging orientations (vertical and horizontal) were evaluated with the four different loop widths (2.0, 2.5, 3.0, and 3.5 mm) and three film support designs. Five different loops for each configuration were used as replicates with 10 testing runs for each loop. The occurrence rate of each vitrification classification was calculated as: (Occurrence times/10) × 100%. The effect of different loop heights (only 0.4 mm was evaluated in this study) was not investigated, because reports have shown that 0.2–0.4-mm loop heights had the highest efficiencies in producing high-quality vitrification, but the printing quality of 0.2-mm loops was not consistent [20].

### 2.5. Evaluation of Volume Capacity

A loop length of 8 mm and width of 3.5 mm was selected for evaluation of the volume capacity. The effects of different film support configurations (open, transverse, and axial) and two different sample loading methods on the volume capacity were evaluated. With a ‘direct submerging’ method, the samples were loaded by the submerging of loops into DI water (>300 μL) in a 1.5-mL centrifuge tube (Figure S3). In situations when the sample volume was too low to submerge loops, an ‘indirect submerging’ method was used. With this method, the samples were loaded by placing a 10-μL drop onto a glass slide, and the loop was placed onto the droplet (Figure S3). The mass of DI water loaded on loops was measured with an analytical balance (Mettler AE 166, Columbus, OH, USA). Five different loops of each configuration were replicates, with five measurements for each loop.

### 2.6. Statistical Analysis

All statistical analyses were performed using SAS 9.4 (SAS Institute, Cary, NC, USA). The chi-square test was used to compare differences in the film retention and vitrification occurrence rates. The volume capacities among the different loop supports were compared by one-way ANOVA (all assumptions met). The volume capacities between the two submerging methods were compared by a *t*-test.

## 3. Results

### 3.1. Design

**Figure 2.**
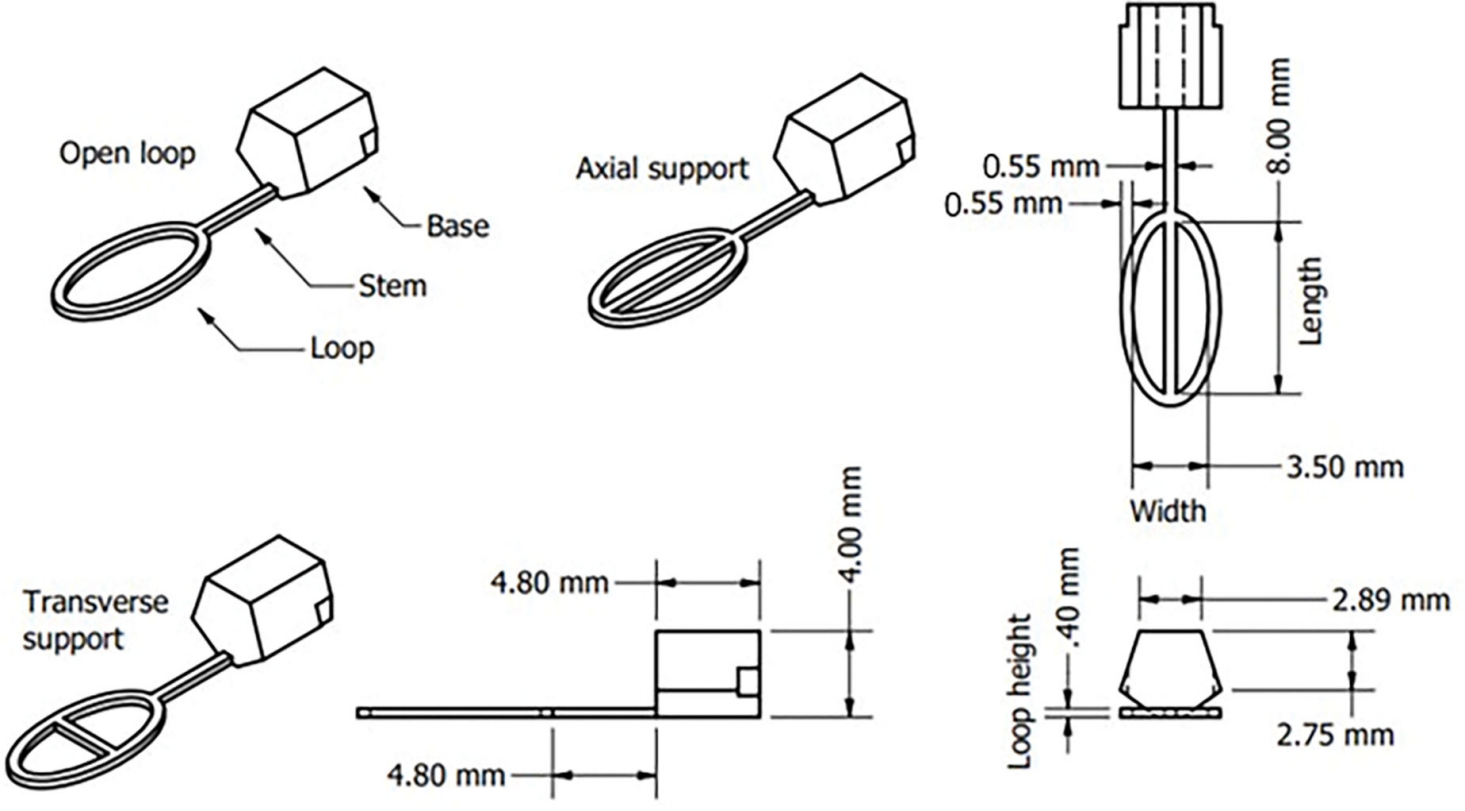
Dimensioned drawings of the 8-mm axial support VDCV. A total of 36 configurations were developed and tested, including three different support configurations (open, axial, and transverse); 3 different loop lengths (8, 10, and 12 mm); and four different loop widths (2, 2.5, 3, and 3.5 mm).

The vitrification component of the VDCV included a base, a stem, and a loop feature (Figure 2). The pentagonal cross-section of the base functioned as a fastening adaptor that could be easily inserted into a vial cap. The stem provided space between the base and the loop to prevent interference with the sample cooling rate by thermal mass contributions of the base. There were three different designs of supporting mechanisms of loops and three different loop lengths and four different loop widths for each supporting mechanism. Loops with different axis lengths had different total lengths with the same length of stems and bases. A loop height of 0.4 mm was chosen based on previous reports noting the optimal FDM printing efficiency [19,20]. The handling component of the VDCV featured a cap adaptor, a shaft, and a base. The cap adaptor (Figure S4) was designed to fasten caps of two types of vials for capping and decapping, with structures on a central pillar to fit Nunc^™^ Bank-It^™^ cryogenic storage vials (0.5 mL) and inside circular wall to fit Corning^®^ cryogenic vials (2.0 mL). The shaft and base were elongated to facilitate safe handling with liquid nitrogen.

### 3.2. Computer Simulation

**Figure 3.**
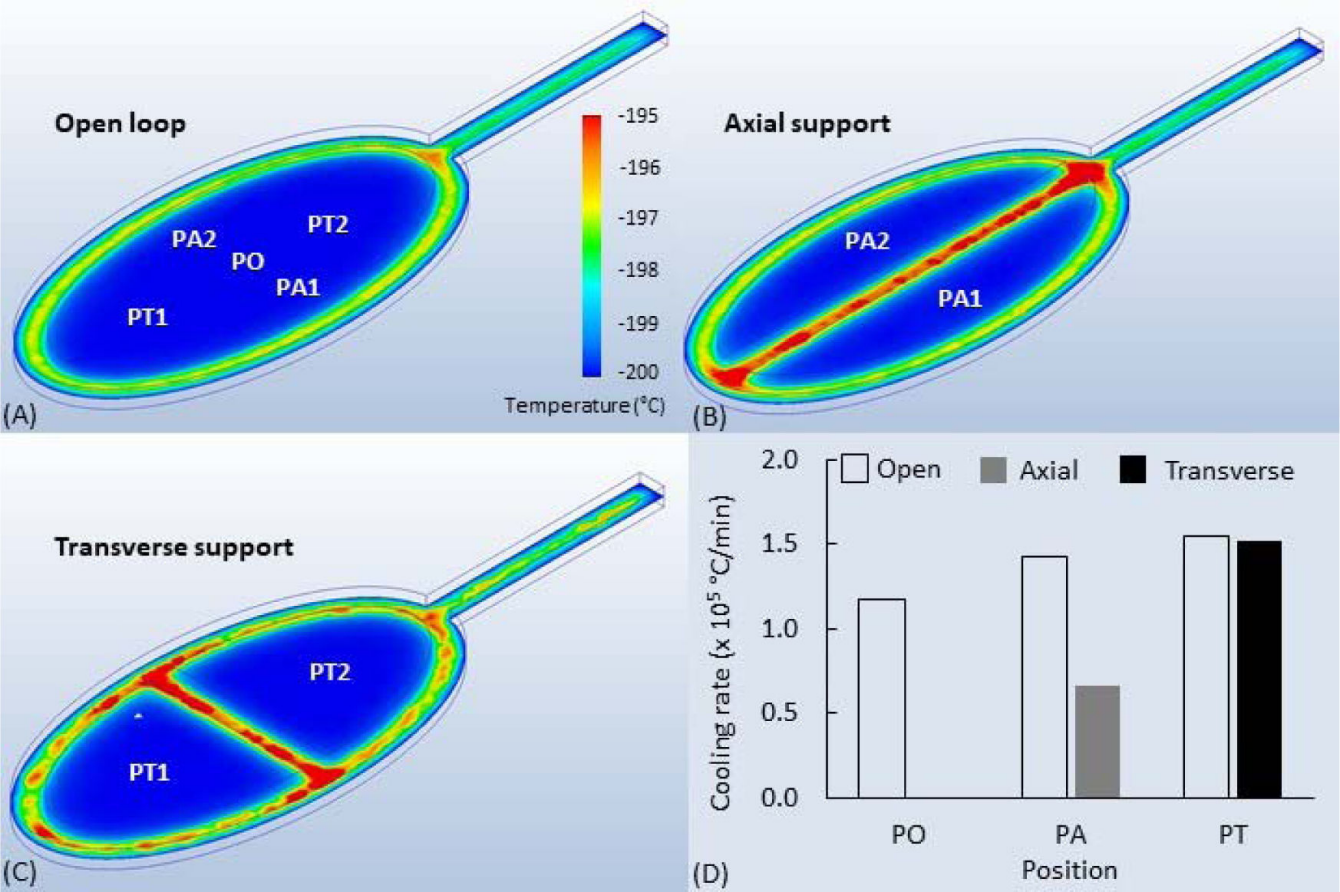
Computer finite element simulation to characterize the temperatures of water films in different VDCV support configurations. Temperature profiles at 1 s after exposure to liquid nitrogen (−200 °C) for (**A**) an open loop, (**B**) loops with axial support, and (**C**) loops with transverse support. (**D**) Comparison of the cooling rates produced in different configurations. Cooling rates of 5 positions in the open loop design were sampled, including the center position for open loops (PO), centers of the two compartments divided by axial supports (PA1 and PA2), and centers of the two compartments divided by transverse supports (PT1 and PT2). The cooling rates were averaged for PA1 and PA2 as PA and PT1 and PT2 as PT.

The computer simulation results indicated that the cooling rates of the points (Figure 3A–C) inside the water films on the open loops ranged from 1.2 to 1.5 × 10^5^ °C/min (Figure 3D). The cooling rates of the sample points for the loops with transverse supports were comparable to the open loop, whereas the sampling points on the loops with axial supports had a 50% slower cooling rate (Figure 3B–D). At 1 s following exposure to −200 °C (Figure 3A–C), the water samples reached an equilibrium liquid nitrogen temperature, while the plastic loop materials were warmer by several degrees Celsius. At 0.1 s (Figure S5), the central areas of water films were cooled more quickly (about −120 to −160 °C) than the surrounding areas (about −70 to −100 °C) that were closer to the plastic loop material. Films on open loops and transverse supports were cooler faster than those on axial supports (Figures S5 and S6). The base cooled slower because of the larger thermal mass (Figure S7), but it appeared not to slow the sample cooling within the loop when the same simulations were run with and without the base.

### 3.3. Film Retention Prior to Freezing

**Figure 4.**
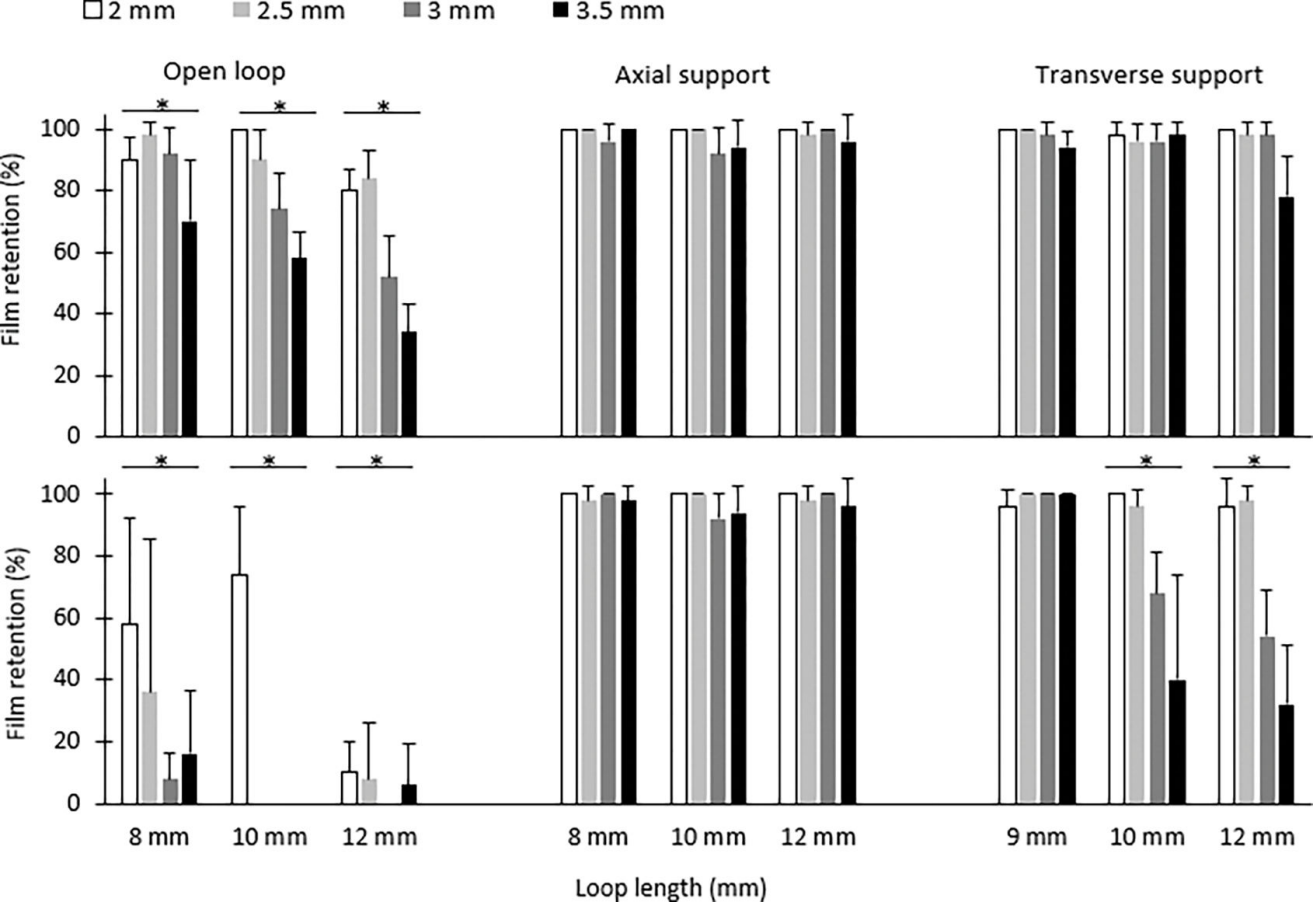
Film retention of water samples prior to exposure to liquid nitrogen. The VCVD was positioned horizontally (**upper**) and vertically (**bottom**) for different support configurations, loop lengths, and loop widths. Error bars represent the standard deviation. Asterisks indicate significant differences among different loop lengths.

Film retention prior to freezing is essential for vitrification. Two general trends were observed (Figure 4): samples held horizontally had overall better film retention than those held vertically in open loops and loops with transverse support. Loops with supports had better film retention than those without supports. With transverse support, no significant differences in film retention were observed among different loop lengths with horizontal holding (*p* > 0.05), whereas decreases in film retention were observed with longer and wider loops when held vertically (*p* < 0.05). All loops with axial support had a film retention > 92% and had no significant differences in film retention among loops with different loop lengths, loop widths, or horizontal versus vertical orientations of the holding angles (*p* > 0.05).

### 3.4. Vitrification

**Figure 5.**
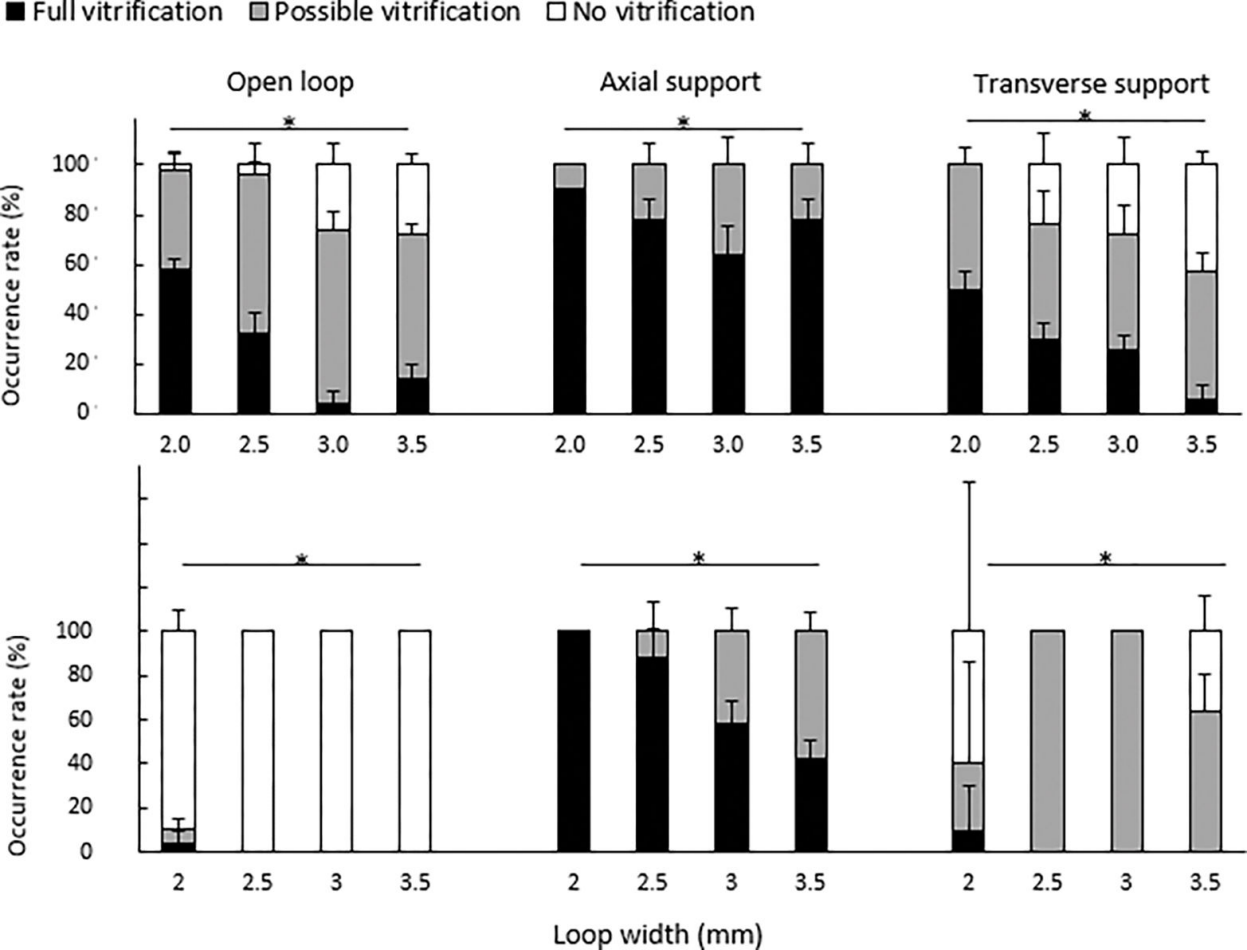
Occurrence rate of vitrification outcomes for different support configurations and loop widths of VDCV held horizontally (**upper**) and vertically (**bottom**). Error bars represent the standard deviation. Asterisks indicate significant differences in full vitrification among different loops widths.

With a fixed 10-mm loop length, the overall trends (Figure 5) of the fully vitrified occurrence rates were: horizontal plunging > vertical plunging and axial support > transverse support and open loop. Only possible vitrification and full vitrification were observed in loops with axial support with any of the plunging orientations tested. The occurrence of full vitrification decreased with the loop width in all the plunging orientations and support configurations.

**Figure 6.**
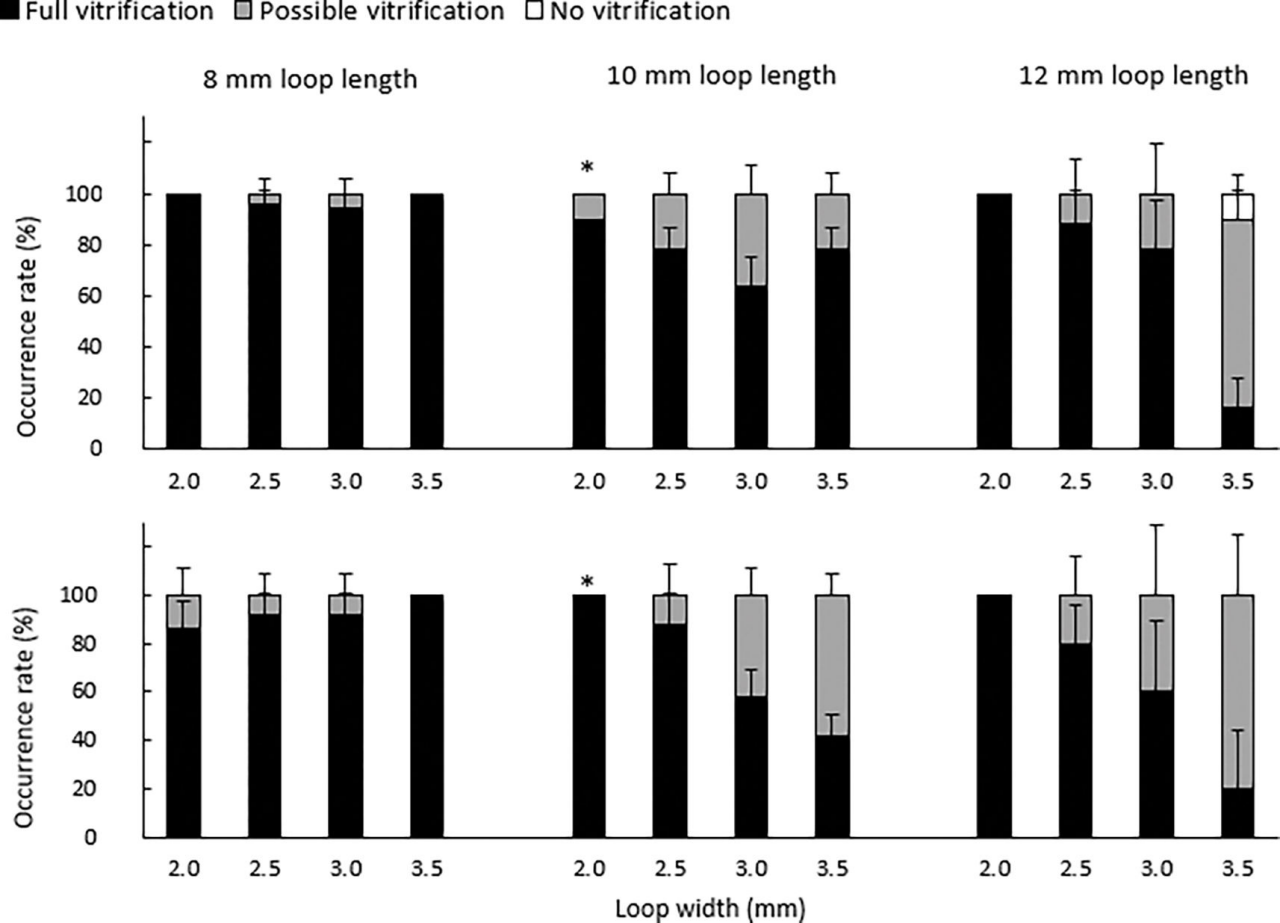
Occurrence rate of vitrification outcomes for different loop widths and lengths of VDCV with axial supports held horizontally (**upper**) and vertically (**bottom**). Error bars represent the standard deviation. Asterisks indicate significant differences in full vitrification between the horizonal and vertical orientations.

Based on this experiment, loops with axial supports were chosen to examine the effects of loop length on the vitrification quality (Figure 6). Loops with an 8-mm loop length produced 86–100% of the full vitrification in all the loop widths and plunging angles. A significant difference (*p* = 0.001) in full vitrification was observed between vertical and horizontal plunging in loops of 10 mm × 2.0 mm. The lowest occurrence of full vitrification (16–20%) was observed in the longest (12 mm) and widest (3.5 mm) loops, regardless of the plunging orientation. The prototypes were visually examined after thawing, with no physical damages observed.

### 3.5. Volume Capacity

**Figure 7.**
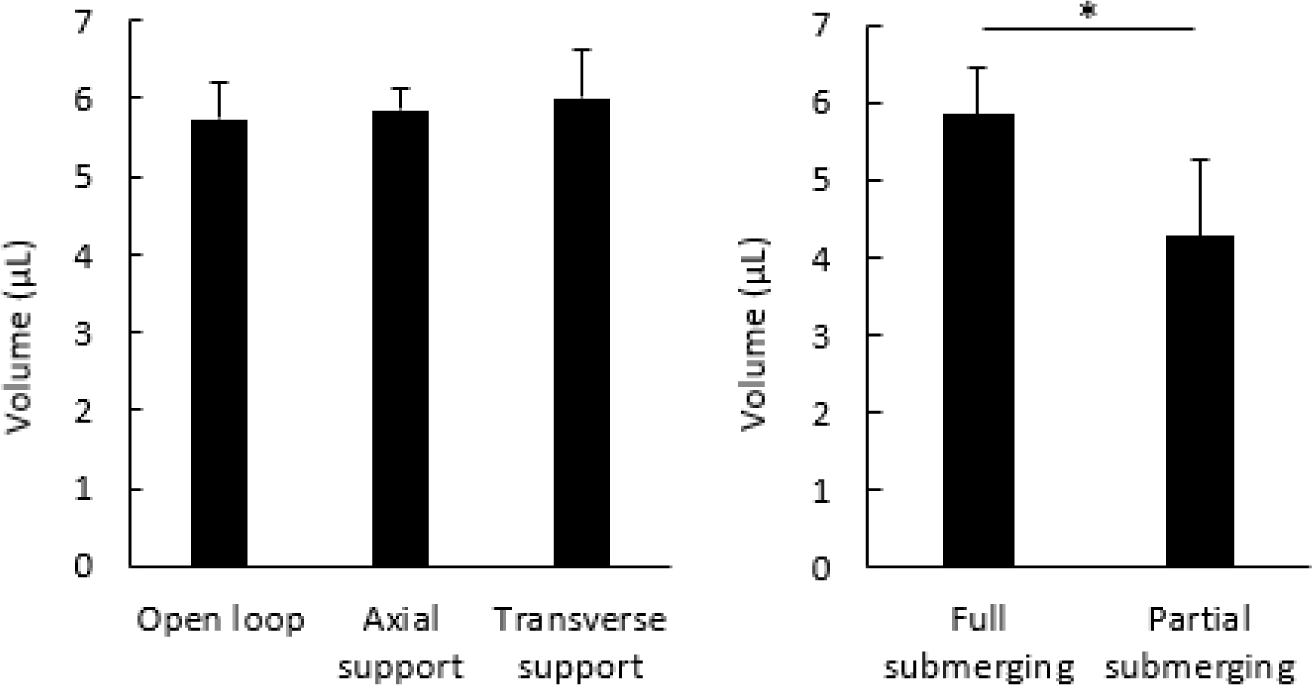
Volume capacity of the vitrification component with a loop length of 8 mm and width of 3.5 mm with different support configurations (**left**) and loading methods (**right**). Error bars represent the standard deviation. Asterisk indicates significant differences among the groups.

It is important to evaluate if the addition of support structures causes a reduction of the capacity volumes. Based on previous experiments, loops with 100% vitrification occurrence (length of 8 mm and width of 3.5 mm) were selected for volume capacity evaluation. No significant differences (*p* = 0.6584) were shown in the volume capacity (5.7–6.0 μL) among the three supporting methods (Figure 7). The volume capacity by indirect submerging was 25% smaller (*p* = 0.0359) than that of direct submerging (Figure 7).

## 4. Discussion

Sperm cryopreservation by vitrification offers several advantages over equilibrium freezing (slow cooling), including: (1) lower cost (no programmable freezers are needed [5], (2) higher portability (no need to transport freezers and pressurized liquid nitrogen tanks), and (3) faster processing time (plunging in liquid nitrogen within seconds). Although the small volumes can limit the usage of vitrification in animals that produce large numbers of oocytes, it has great potential for small-bodied species with miniscule sperm volumes [24]. For example, among fishes, swordtails and guppies (family Poeciliidae) are useful biomedical models and popular ornamental aquaculture species [25] and typically provide <5 μL of sperm from each male [26,27]. Zebrafish (*Danio rerio*) is another important biomedical research model, and they typically provide <1 of μL sperm per male [28]. A lack of standardized devices has impeded the application of vitrification in sperm cryopreservation and repository development, because divergent procedures and protocols developed for various tools result in low reproducibility [20]. In addition to the vitrification process itself, appropriate storage and sample identification are essential elements of sample management for applied germplasm repositories. Vitrification devices developed in the present study can be accessed and distributed by open-sharing platforms to allow customization and eventual standardization among user communities.

### 4.1. Design

The VDCD prototypes were designed to integrate with existing cryopreservation vials with secure storage and identification functions. The 0.5-mL Bank-It^™^ cryogenic storage vials are used for zebrafish sperm cryopreservation because of a reliable 2D barcode identification system, compatibility with standard microplate racks and cryo-boxes, capability of automated capping (and decapping) systems, and compatibility with the recommended protocols [29]. In previous reports, 3D printed vitrification devices were developed to fit inside protective sleeves (inner diameter of 3.1 mm) with a 1.7-mm loop width [20]. In this study, to take advantage of the larger internal space of cryo-vials (inner diameter of 6.7 mm), wider loops were designed (2.0–3.5 mm) that could hold larger volumes (6 uL vs. 2 uL of loops that could achieve vitrification in the previous reports). A major design feature of the VDCD was the addition of loop supports to address sample film instability caused by wider loops. In future studies, larger loops can be designed for different types of vials, and more varied supporting structures could be evaluated based on the present study. A base was designed to secure frozen samples without contacting vial walls in case of movement caused by the sample transfer (or transportation). The handle with capping and decapping functions was operated manually in this study, but it can be used as a basis for the development of an automated high-throughput VDCD that is able to cap and decap multiple vials in batches.

### 4.2. Computer Simulations

Thermal finite element computer simulations can facilitate the design process of devices for cryogenic applications. With computer simulations, promising candidates can be identified within several days (depending on the computing time with average convergence time of ~2 h in the present study) instead of the weeks that would be needed for the fabrication and empirical evaluation of multiple prototypes to identify the optimal geometries. To the best of our knowledge, no computer simulations of cooling rates for sperm vitrification devices have been previously reported. The cooling rates of 0.7–1.5 × 10^5^ °C/min were within the temperature range (>2000 °C/min) of vitrification occurrence [4]. When compared with previous numerical simulation studies of various oocyte vitrification devices [12], the cooling rates in the present study were higher than the open-pulled straw and pipette tip methods (1.7–8.7 × 10^4^ °C/min) comparable to Cryotop^®^ (1.0–3.7 × 10^5^ °C/min) and lower than Cryoloop^®^ (3.6–18 × 10^5^ °C/min). Cryotop^®^ was created to vitrify embryos on a thin plastic film that has subsequently been adapted for research to hold droplets of sperm suspension for vitrification but with a relatively high cost (i.e., ~$20/unit vs. $0.5/unit material cost of VDCD). The mechanism of enabling vitrification by the VDCD were similar to those of the Cryoloop^®^ by forming thin films to increase the cooling rate. The higher apparent cooling rate of devices such as Cryoloop^®^ could be caused by the smaller sample size (i.e., <1 μL) and minimal supporting materials (i.e., <20-μm height). The lower cooling rate of loops with axial supports could be caused by larger thermal masses of the central support. The purpose of the computer simulations herein was to compare the contributions of various geometries on the relative loop cooling rates. An exact determination of the actual temperature profiles in these PLA loops would need to consider several of the polymer physicochemical properties that have not been reported and was thus beyond the scope of this study.

### 4.3. Film Retention

Ideally, sample films should be stable to avoid repeated loading, which will reduce the efficiency and potentially waste valuable samples. The effects of handling methods of different loop configurations on film retention prior to vitrification were not previously reported. In the present study, loops held horizontally showed a higher film retention rate than those held vertically in open and transverse loops, presumably resulting from fluid being pulled downward due to gravity. Generally, loop supports increased the film stability, and axial supports exhibited the best film retention. This could be caused by the narrower space among supporting structures provided by axial supports to stabilize films. A film failure that originates at the loop edge may depend on the interfacial tension between the liquid and air, as well as the liquid and the PLA. At the edge of the loop, there is a three-phase interface (solid, liquid, and gas) where the film behavior would be complex to simulate. Due to its lower viscosity than most vitrification solutions, water was used in this study to represent a conservative model for film retention and, also, to facilitate standard comparisons across other studies [20]. However, cryoprotectants (e.g., dimethyl sulfoxide, ethylene glycol, and glycerol) [2] with higher viscosities will be used for vitrification in actual applications [30] and, thus, likely increase the film retention compared with the observations herein.

### 4.4. Vitrification

Two factors are essential to achieve sample vitrification: a sufficient film stability and high cooling rate. Film failure could occur during handling prior to freezing (as discussed above) or during freezing caused by the unstable environment resulting from liquid nitrogen phase changes [31]. The trends of horizontal > vertical and axial > open and transverse loops in full vitrification could be caused by a higher film stability provided by horizontal handling and axial support that were also observed in the film retention experiment. Generally, loops with lower lengths and widths had higher vitrification occurrence, which was consistent with the previous reports [19,20]. This trend could be explained by a higher cooling rate due to the smaller sample volumes in these designs or less insulation by the loop material. Sample volume and viscosity are two major factors that affect the vitrification [32,33]. In the present study, the sample viscosity was not varied and evaluated but could be an interesting topic to investigate in future studies.

### 4.5. Volume Capacity

The volume capacity has a positive correlation with the dimensions of vitrification loops, such as length and height [20], and thus, this correlation evaluation was not included in this study. The addition of loop supports did not affect the volume capacity. This could result from a reduction of the area of the central (thinner) films that occurred in open loops. The effects of multiple supporting structures on the volume capacity should be further studied. The sample shapes within the loop are dependent upon several factors, including the physical properties of the sample liquid and the loop polymer materials and their interactions. For example, due to the surface tension, there are meniscus-like curvatures [34] of the film that are dependent upon loop geometries that would result in different volumes for different support configurations. The sample volume loaded by indirect submerging was 25% lower than those loaded by direct submerging, indicating that the handling method could affect the volume capacity. However, partial submerging has its advantages when working with small-bodied species, because sample volumes of <30 μL do not allow the full submerging of loops.

The biological context of the loop sample volume capacity is important to consider when optimizing vitrification procedures. A sperm sample volume of 0.5–1.0 μL (~2 × 10^9^ cells/mL) can be collected from individual zebrafish by stripping [29,35]. In the established protocol for equilibrium freezing from ZIRC, about 20 μL of sperm diluted to 1–5 × 10^8^ cells/mL are frozen in each 0.5-mL vial, and 10–20-μL of thawed sperm are used to fertilize about 300 eggs from individual females [29]. This provided ratios of 1–2 × 10^7^ sperm/vial, 1 to 2 males/vial (based on the 1 × 10^8^ cells/mL cooling concentration), and 300–600 eggs/vial. Upon collection, sperm can be diluted to 1 × 10^9^ cells/mL for vitrification [2], with a diluted volume of 1–4 μL. It is necessary to point out that the sperm concentration for vitrification is higher than those used in equilibrium freezing [36,37]. Based on a volume capacity of 5.7–6 μL (for 8 mm × 3.5 mm-loops), a single unit of VDCV can provide about 6 × 10^7^ sperm/vial, pooling samples of 3–6 males/vial, and fertilization for 1800 eggs/vial. These loading efficiencies are comparable or better than the existing protocols [29]. Fertilization experiments can be conducted in the future to confirm this estimation.

## 5. Conclusions

In this study, prototypes of a low-cost (US$0.5 material cost), portable, customizable, 3D printed vitrification device compatible with cryopreservation vials were developed. These were composed of a loop and a handle to provide the functions of sample loading, vitrification, storage, and identification. Loops with 8-mm lengths and axial supports produced 86–100% full vitrification occurrences in all the loop widths and plunging orientations. A single unit of VDCV can provide efficiencies of about 6 × 10^7^ sperm/vial, pooling samples of 3–6 males/vial, and fertilization for 1800 eggs/vial. The VDCV prototypes can be accessed by user communities through open file sharing and be fabricated with consumer-level 3D printers, thus facilitating community-level adoption. In this approach, user communities can become maker communities, producing inexpensive standardized devices distributed as open scientific hardware [38,39]. These prototypes can be used as the basis for future studies in design improvement and biological testing. Although the CDVC was developed initially for aquatic biomedical species, it can be potentially used for other animals with small sperm volumes, such as amphibians, reptiles, birds, and small-bodied mammals.

## Supplementary Material

Stencil.stl

Stylus.stl

Dock.stl

Loop1

Loop2

Loop3

Loop5

Loop4

Loop6

Loop7

Loop8

Loop9

Loop10

Loop11

Loop12

Loop15

Loop13

Loop14

Loop16

Loop17

Loop18

Supplementary Material
