# Supplementary material for: A 3D Printed Vitrification Device for Storage in Cryopreservation Vials
Source: Appl Sci (Basel). Author manuscript; Available in PMC 2023 Feb 9. (PMC9910574; doi:10.3390/app11177977)
Supplement: Supplementary Material [file NIHMS1863302-supplement-Supplementary_Material.pdf]

Supplemental Materials

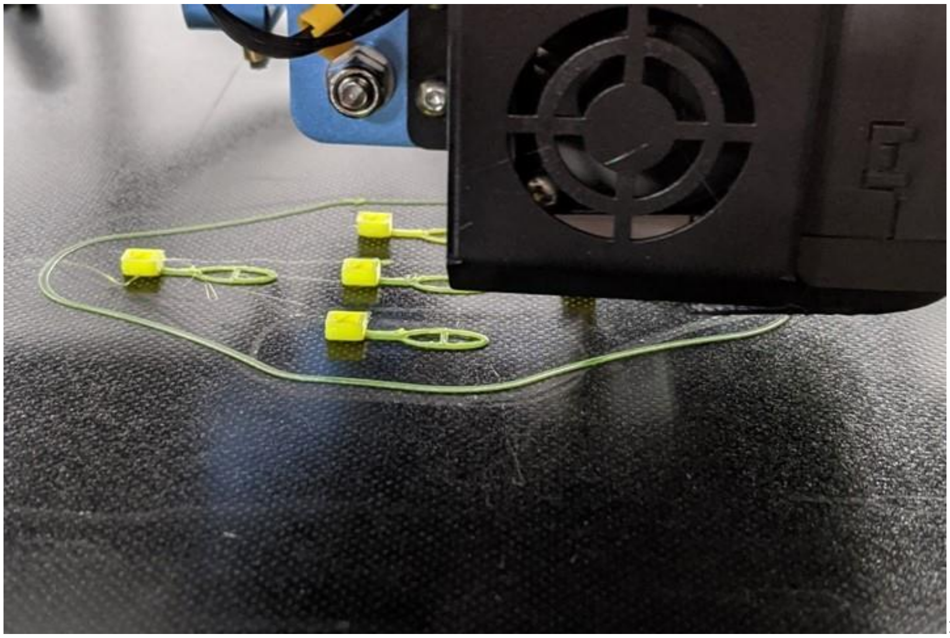:

Figure S1: Fabrication of a batch of 5 vitrification components of Vitrification Device for Cryo-Vials (VDCV) prototypes by 3D printing. Lines of filament material surrounding the pro-totype objects are 'skirts' that facilitate printing initiation.

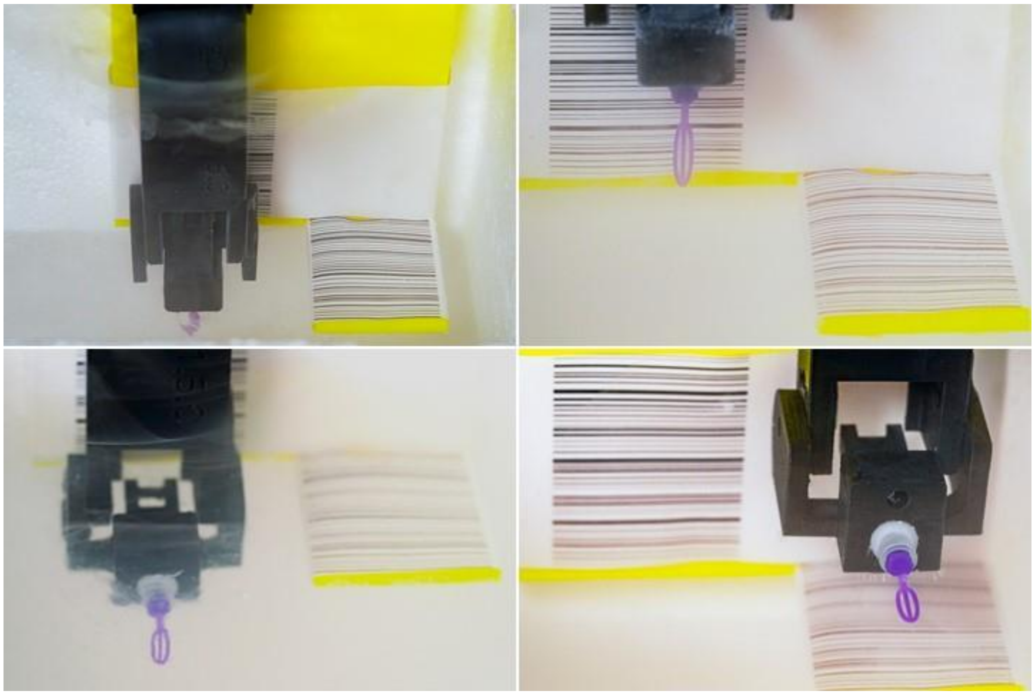

Figure S2: Evaluation of vitrification with different orientations. The VDCV were (A) vertically plunged into liquid nitrogen and (B) assessed in front of an evaluation station and horizontally (C) plunged and (D) assessed.

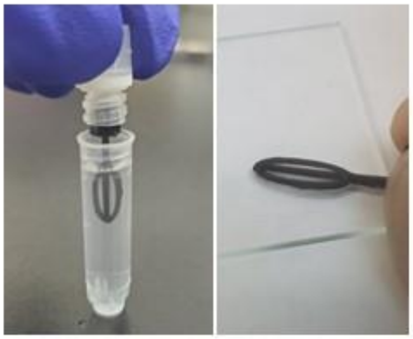

Figure S3: Loading of samples into the VDCV with direct submerging (left) and indirect submerging (right).

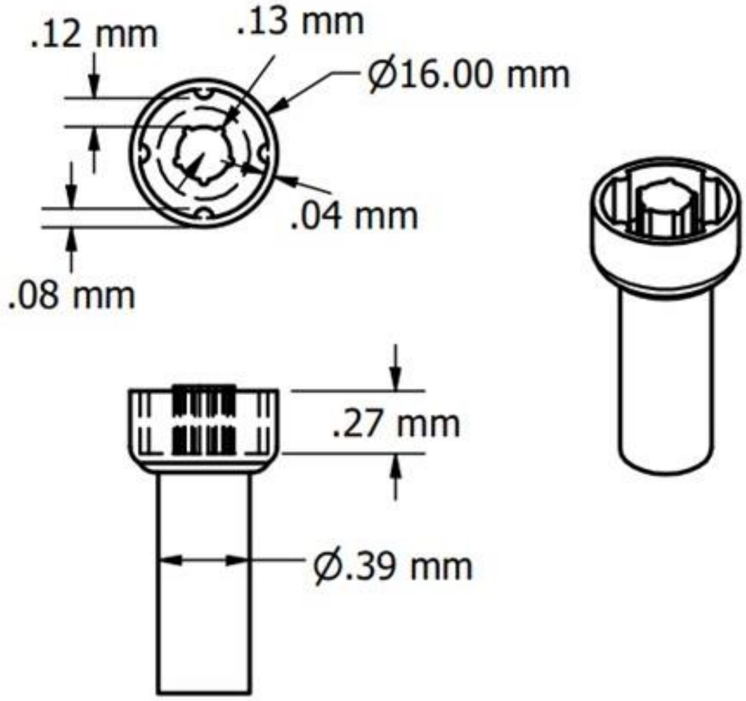

Figure S4: Dimensioned drawings of the cap adaptor feature of the handling component of VDCV.

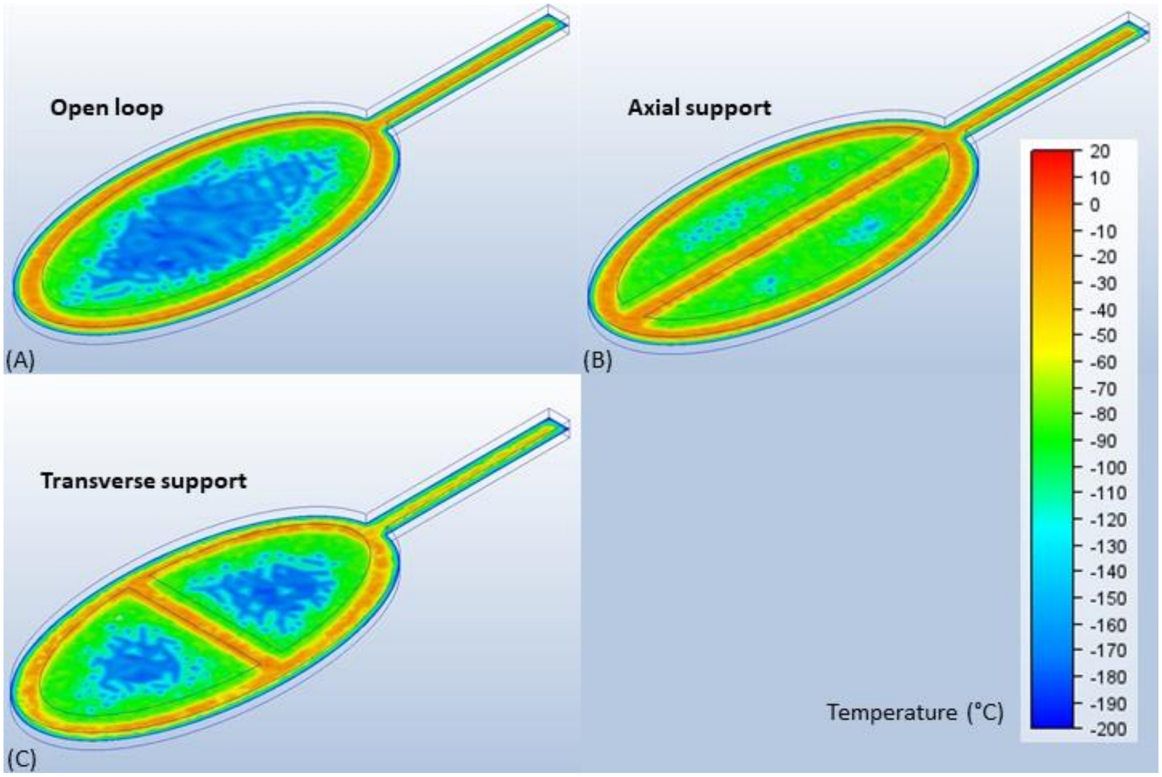

Figure S5: Computer simulation to characterize the temperature of the water film suspended in prototypes of the VDCV. Temperature profiles at 0.1 s after exposure to liquid nitrogen (−200 °C) for (A) the open loop, (B) loops with axial support, and (C) loops with transverse support.

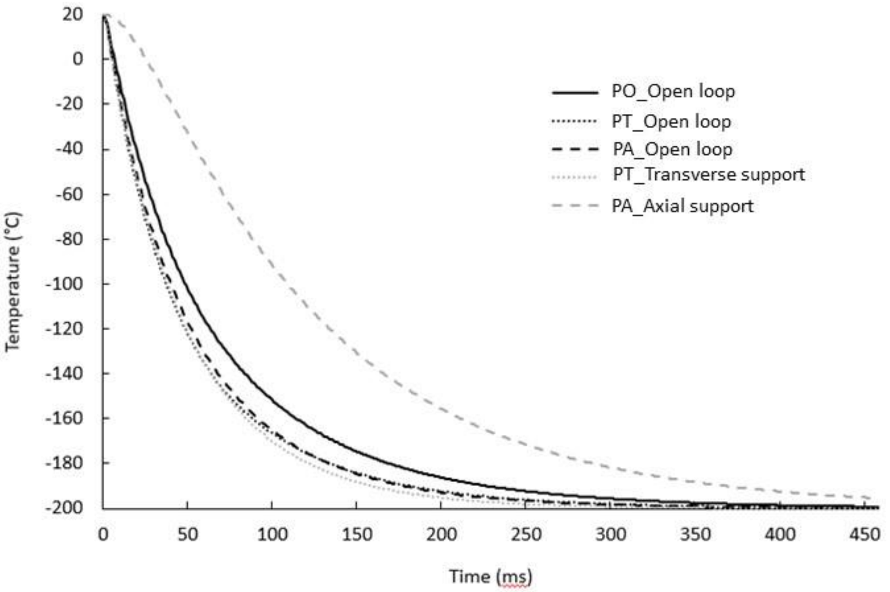

Figure S6: Temperature profiles of locations within water samples in VDCV loops with different support configurations. Cooling rates of 5 positions (shown in Figure 3) in the open loop design were sampled, including a center position for open loops (PO), centers of the two

compartments divided by axial supports (PA1 and PA2), and centers of the two compartments divided by transverse supports (PT1 and PT2). The cooling rates were averaged for PA1 and PA2 as PA and PT1 and PT2 as PT.

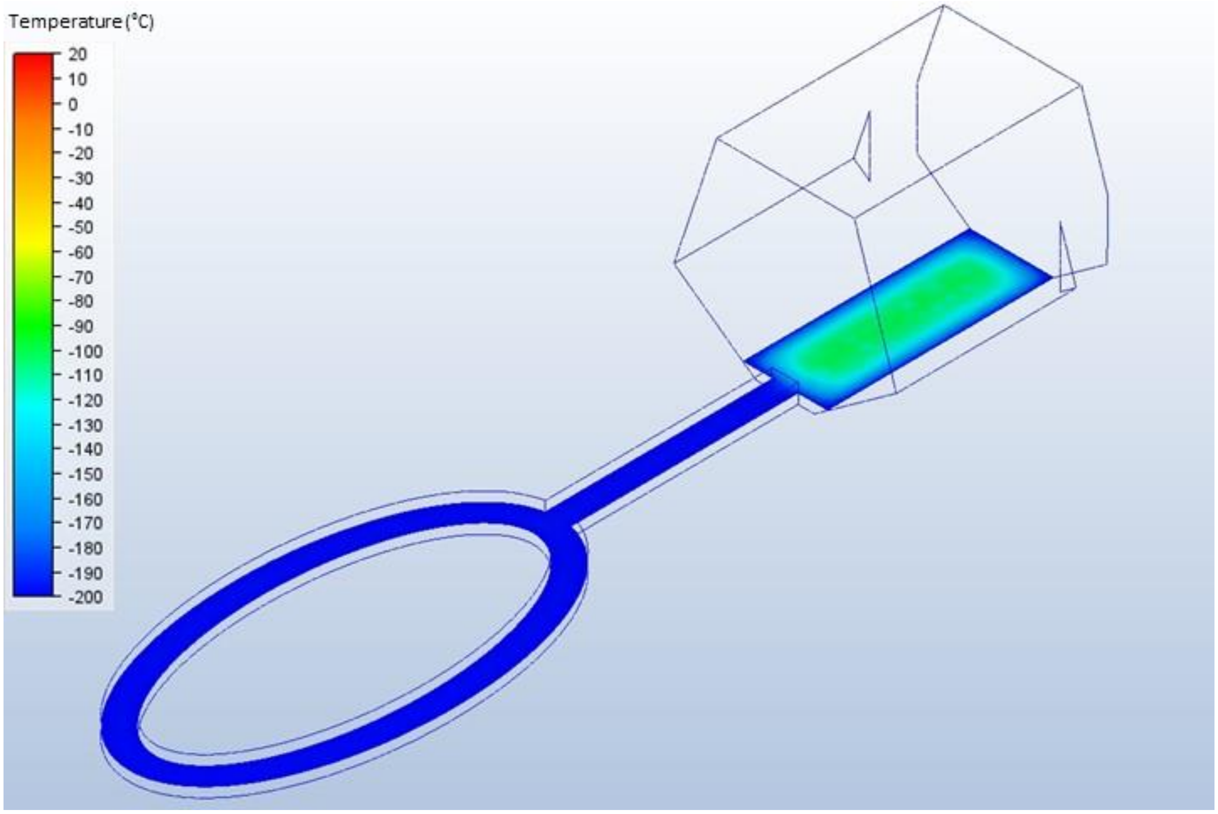

Figure S7: Computer simulation to characterize the temperature of thermoplastic to fabricate the vitrification component of the Vitrification Device for Cryo-Vials (VDCV). The temperature profiles at 1 s after exposure to liquid nitrogen (−200 °C) indicated the base cooled slower than the loop.

Supplemental Table S1. Specifications for 3-D printing of prototypes of Vitrification Device for Cryopreservation Vials.

Parameters	Specifications
Printer name	Sovol SV01 3D Printer
Slicing software	Ultimaker Cura Version 4.6
Filament manufacturer	ZYLtech Engineering (TX, USA)
Filament material	Polylactic acid
Filament diameter	1.75 mm
Hotend temperature	200 °C
Print speed	50 mm/s for infills and outer layers
Nozzle diameter	0.4 mm
Nominal layer height	0.2 mm
Retraction distance	5 mm
Retraction speed	50 mm/s

Print bed temperature	60 °C
Build surface material	Tempered Glass (300mm x 255mm)
Part cooling fan speed	100%
First layer printing speed	5 mm/s
Infill rate	40%
Infill pattern	Zig Zag
Perimeter layer number	2
Top layer number	2
Bottom layer number	2
Support usage	No applied
Build volume	11.2″ L × 6.0″ W × 6.1″ H
